# FT/TFL1: Calibrating Plant Architecture

**DOI:** 10.3389/fpls.2019.00097

**Published:** 2019-02-13

**Authors:** Tatiana Souza Moraes, Marcelo Carnier Dornelas, Adriana Pinheiro Martinelli

**Affiliations:** ^1^ Laboratório de Biotecnologia Vegetal, Centro de Energia Nuclear na Agricultura, Universidade de São Paulo, Piracicaba, Brazil; ^2^ Departamento de Biologia Vegetal, Instituto de Biologia, Universidade Estadual de Campinas, Campinas, Brazil

**Keywords:** *FT/TFL1*, model plant, *Passiflora*, plant architecture, tendril

## Abstract

There is a very large diversity in plant architecture in nature. Over the past few years, novel theoretical concepts and analytical methods have emerged as powerful tools to understand important aspects of plant architecture. Plant architecture depends on the relative arrangement of three types of organs: leaves, shoots, and flowers. During plant development, the architecture is modulated by the balance of two homologous proteins: FLOWERING LOCUS T (FT) and TERMINAL FLOWER 1 (TFL1). The FT/TFL1 balance defines the plant growth habit as indeterminate or determinate by modulating the pattern of formation of vegetative and reproductive structures in the apical and axillary meristems. Here, we present a summarized review of plant architecture and primarily focus on the *FT/TFL1* balance and its effect on plant form and development. We also propose passion fruit as a suitable model plant to study the effect of *FT/TFL1* genes on plant architecture.

## Introduction

Our understanding of plant architecture has advanced in the last few decades, and research in this field has given rise to innovations in various aspects of plant science. The use of high-performance computers for plant growth data analysis and simulation has contributed to the development of various interpretations of plant architecture (Kuchen et al., 2012; Coen et al., 2017; Whitewoods and Coen, 2017).

Plant architecture is determined by the number and arrangement of organs that are formed from the shoot apical meristem (SAM) (Benlloch et al., 2007). During the vegetative stage, the SAM gives rise to shoots and leaves, and after transition to the reproductive stage it produces flowers (Benlloch et al., 2007).

In the annual model plant *Arabidopsis thaliana*, the growth habit is monopodial and the apical meristem remains indeterminate and active throughout the entire plant life cycle (Bowman, 1994). The resulting stem bears lateral branches, leaves, and flowers, and there is a clear distinction between the vegetative and reproductive stages (Bradley et al., 1997).

Perennial plants differ from annual herbaceous plants, such as *Arabidopsis,* in a range of characteristics that influence their growth pattern and consequently the plant architecture. The branching habit of a perennial plant is more complex because an axillary meristem can have multiple fates—it either directly forms a shoot, or differentiates into a floral bud that opens the following spring after a dormant period, or remains dormant indefinitely. In addition, in perennial plants, the SAM preserves a high level of vegetative identity or “vegetativeness” (Prusinkiewicz et al., 2007). According to these authors, the meristem will form either a flower or a branch depending on its “vegetativeness”; high “vegetativeness” corresponds to an indeterminate shoot growth, and low levels of “vegetativeness” lead to determinate growth and development of floral meristem (Prusinkiewicz et al., 2007).

Plant architecture is controlled by genetic mechanisms associated with environmental factors and largely dependent on meristem identity, which establishes the development of shoots or flowers. Extensive studies on genetic mechanisms controlling meristem identity in *Arabidopsis* have revealed that plant architecture is regulated by a few groups of genes (Bradley et al., 1997; Conti and Bradley, 2007; Ho and Weigel, 2014). Among those, we can highlight *FLOWERING LOCUS T* (*FT*) and *TERMINAL FLOWER 1* (*TFL1*), both belonging to the *FT/TFL1* gene family and encoding proteins similar to phosphatidylethanolamine binding proteins (PEBP) (Wickland and Hanzawa, 2015). The balance between these two homologous proteins, FT and TFL1, controls the indeterminate and determinate growth in plants and modulates plant architecture, regulating the formation pattern of vegetative and reproductive organs from the apical meristem (Park et al., 2014).

In the present paper, we report an updated view on the modulation of axillary meristems and plant architecture, with a primary focus on the role of *FT/TFL1* genes. We introduce new discussions about the current knowledge in this field and the possible implications and perspectives concerning plant architecture in plant developmental studies.

## Effects of *FT/TFL1* Balance in Annual Plants: *Arabidopsis*

In *Arabidopsis*, six genes have been identified in the *FT/TFL1* family*: FLOWERING LOCUS T* (*FT*) and *TWIN SISTER OF FT* (*TSF*), involved in flowering promotion and belonging to the *FT*-like subfamily; *TERMINAL FLOWER 1* (*TFL1*), *BROTHER OF FT AND TFL1* (*BFT*) and *Arabidopsis thaliana CENTRORADIALIS HOMOLOG* (*ATC*), involved in flowering repression and belonging to the subfamily *TFL1*-like; and *MOTHER OF FT AND TFL1* (*MFT*), belonging to the *MFT*-like subfamily and involved in the regulation of seed germination (Kobayashi et al., 1999; Xi et al., 2010; Wickland and Hanzawa, 2015).

FT and TFL1 have antagonistic functions in plant development. Considered as the florigen agent, FT activates the flowering pathway, whereas TFL1 represses flowering and is responsible for the maintenance of the inflorescence meristem. The FT/TFL1 balance modulates the plant architecture because both proteins are involved in the control of the indeterminate versus determinate plant growth habit, which is essentially based on the production pattern of vegetative versus reproductive organs by the apical meristem (Matsoukas et al., 2012; Xu et al., 2012; Jaeger et al., 2013; Nakano et al., 2015; Patil et al., 2017).

In *Arabidopsis*, the transcription factor CONSTANS (CO) activates *FT* in the leaves, where the gene is transcribed and translated, and its protein is then transported *via* phloem into the vegetative apex. In the apex, the FT protein forms a complex with a bZIP protein, FLOWERING LOCUS D (FD). This complex activates genes involved in floral meristem identity, such as *LFY* and *APETALA1*, thereby inducing flowering (Abe et al., 2005). The *ft* mutants flower late and present indeterminate growth, whereas the overexpression of *FT* causes early flowering and conversion of the SAM into a terminal flower (Corbesier et al., 2007). In contrast, the expression of *TFL1* in the SAM maintains the indeterminate growth and represses the floral meristem identity genes. The TFL1 protein is also capable of interacting with the FD transcription factor. Thus, *tfl1* mutants flower early and their SAM is converted into a terminal flower. In contrast, overexpression of *TFL1* causes late flowering and prevents the formation of a terminal flower (Bradley et al., 1997).

## Effects of *FT/TFL1* Balance in Perennials: Tomato

In tomato (*Solanum lycopersicum*) the balance between *FT* and *TFL1* orthologs *SINGLE FLOWER TRUSS* (*SFT*) and *SELF-PRUNING* (*SP*), respectively, coordinate the primary growth with regular sympodial cycles. A high *SFT*/*SP* ratio in the meristem promotes determinate growth, eventually converting the SAM into a flower, while a low *SFT*/*SP* balance promotes indeterminate plant growth (Pnueli et al., 1998, 2001; Lifschitz et al., 2014).

Studies have shown that *sft* mutations may increase the productivity of tomato plants through a determinate growth habit (Park et al., 2014). In *sft*, the loss of florigen activity results in a highly vegetative plant with fewer flowers and fruits. When plants with a determinate growth are heterozygous for *SFT*, there is a partial reduction of florigen activity and a slight suppression of SP, resulting in more sympodial branches and inflorescences. In contrast, when *SP* is present as a dominant allele, plants show indeterminate growth and continuous formation of inflorescences and fruits. Nonetheless, when the tomato plant has a recessive allele for this gene, it exhibits a specific architecture characterized by an early interruption of inflorescence production and shorter plant stature (Pnueli et al., 1998, 2001; Jiang et al., 2013). These results suggest that *sft* and *sp* mutations combined with heterozygous dosage effects should be further explored to modulate flowering and plant architecture and optimize tomato yields.

## How *FT*/*TFL1* Gene Duplication Contributes to the Evolution of Plant Architecture

Gene duplication, a process that gives rise to paralogs, is a very common phenomenon in plants and an important source of new adaptive functions prone to selection during evolution (Kondrashov et al., 2002). Some gene pairs formed by duplication might have a short lifetime—only one copy might be kept functional, while the other copy is pseudogenized—but other gene pairs might persist after duplication. Paralog proteins may give rise to new functions through mutations that affect, for example, gene expression or amino acid sequences, resulting in different phenotypes that arise through adaptive evolution of new protein functions (Lynch and Conery, 2000).

Apparently, during evolution, some *FT* homologous genes acquired the function of flowering suppression. In some species, there is an *FT* with a repression function that antagonizes the flowering induction function of its paralog (Kotoda et al., 2010; Pin et al., 2010; Hsu et al., 2011; Harig et al., 2012). It is of great significance that the evolution of *FT* paralogs might represent a common strategy in plants to refine floral initiation according to multiple environmental and endogenous pathways intrinsic to each individual.

In *Beta vulgaris*, the regulation of flowering time is controlled by BvFT1 and BvFT2, which show high sequence similarity to the *Arabidopsis* FT protein (AtFT). These genes regulate flowering time in response to low temperatures during winter associated with the phenomenon of vernalization. However, these two paralog genes in beet have antagonistic functions. While *BvFT2*, which is functionally conserved, is essential for flowering (it is expressed late in the afternoon, in long days), BvFT1 represses the flowering (it is preferentially expressed early in the morning, in short days) (Pin et al., 2010). Pin et al. (2010) observed that both proteins, BvFT1 and BvFT2, contain amino acids that determine the FT function (Tyr85 and Gln140). However, the binding of specific residues at the external loop of their tertiary structures differed between the two proteins. Thus, these authors suggest that BvFT1 was initially a promoter of flowering, but that mutations within the outer loop of the protein resulted in a change in function toward flowering repression.

Similarly, two FT homolog proteins in *Populus trichocarpa* are required to coordinate the recurrent seasonal flowering cycle in response to temperature (Hsu et al., 2011). PtFT2 is involved in the vegetative growth, and it is activated by high temperatures and long photoperiods during spring and summer. In contrast, PtFT1, which activates reproductive growth, is repressed by high temperatures and induced by winter low temperatures.

Similarly, three out of the four FT homologs identified in *Nicotiana tabacum* repress flowering. Harig et al. (2012) found that all four genes were expressed in leaves under short-day conditions, and at least *NtFT3* expression was restricted to the phloem companion cells. NtFT1, NtFT2, and NtFT3 proteins are floral inhibitors, whereas only NtFT4 is a floral inducer (Harig et al., 2012).

Although *TFL1* gene duplications have also been described in the literature (Carmona et al., 2007; Li et al., 2015), the specific function of each paralog remains unclear, with no reports on *TFL1* paralogs possessing an antagonistic function such as the activation of flowering (Carmona et al., 2007; Li et al., 2015).

## Modulation and Complexity of Axillary Meristems

The axillary meristems (AMs) are important elements in establishing plant architecture and their reproductive success (Wang and Jiao, 2018). The flexibility of the AM activity is directly related to the FT/TFL1 balance (McGarry and Ayre, 2012).

**Figure 1:**
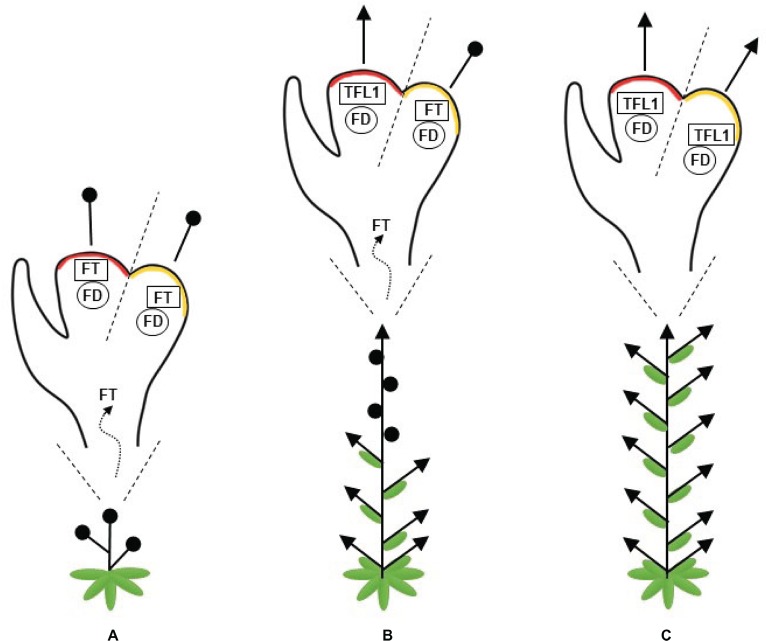
Representation of the changes in plant architecture attributable to the balance between FLOWERING LOCUS T (FT) and TERMINAL FLOWER (TFL1). FT and TFL1 compete for FLOWERING LOCUS D (FD) binding. **(A)** A high FT/TFL1 ratio causes early flowering in plants with short stature, since its apical and axillary meristems are converted into flowers. **(B)** A moderate ratio of FT/TFL1 allows for a balanced development between the shoots and flowers along the plant axes. **(C)** A low FT/TFL1 ratio increases the plant vegetative growth and the apical and axillary meristems give rise to shoots. A red region represents the shoot apical meristem, an orange region represents the axillary meristem, arrows represent indeterminate meristems, and black circles represent flowers.

In summary, a plant with a high FT/TFL1 ratio flowers early and presents a short stature as its apical meristem is converted into a terminal flower. As this ratio decreases, the level of vegetative identity, or “vegetativeness,” increases and the plants produce fewer flowers. Consequently, the repression of *FT* considerably increases vegetative growth (Figure 1).

In most annual plants, the SAM remains indeterminate, while the axillary meristems are determinate. Thus, the SAM gives rise to a vegetative meristem, when FT/TFL1 ratio is low. As the plant ages, FT transport increases because there are more leaves contributing to the FT pool and, in the apex, the effects of accumulated FT exceed the TFL1 function. As a result, a transition from a vegetative to a reproductive meristem is observed and, subsequently, the plant life cycle is completed. In contrast, perennial plants present high levels of TFL1 in the SAM, which remains vegetative, while in the axillary meristems, the FT level prevails, activating genes involved in floral meristem identity (McGarry and Ayre, 2012).

In *Arabidopsis*, the protein encoded by the gene *BRANCHED1* (*BRC1*) interacts with FT, modulating its activity in the axillary buds to repress the premature floral transition of axillary meristems (Hiraoka et al., 2013; Niwa et al., 2013). BRC1, also known as TCP18, is a member of the TCP family, a plant-specific family of transcription factors involved in a large variety of developmental processes, such as cell proliferation and growth, mainly in meristems and lateral organs. Through these processes, it is involved in the establishment of plant form and architecture (Aggarwal et al., 2010; Manassero et al., 2013).

In perennial plants such as lianas, woody climbing vines that are abundant in tropical forests, the growth habit differs. The acquisition of the climbing habit constitutes an innovation, and its success in climbers is related to the development of specialized structures such as tendrils. Lianas begin their life on the floor, but their survival depends on trees for support as they climb upward and compete for sunlight. Thus, their SAM is characterized by indeterminate vegetative growth and repressed development of the AMs, facilitating the lianas to reach the forest canopy (Rodriguez-Ronderos et al., 2016; Sousa-Baena et al., 2018).

The *Arabidopsis* AMs are simple in comparison to AMs in other families such as *Vitaceae* and *Passifloraceae*. Additional accessory meristems, which give rise to tendrils or inflorescences, are a special feature of *Vitaceae*. In grapevine (*Vitis* spp.), a genus of woody perennial vines, adult plants have specific AMs called uncommitted lateral meristems. These meristems are located opposite to the leaves in the expanded shoot and give rise to tendrils for an extended period before the plant initiates flowering. However, upon flowering induction, the inflorescences are formed in place of tendrils from the same uncommitted lateral meristems (May, 2004; Carmona et al., 2008).

In *Passiflora* species, AMs acquire different features during life stages. Taking passion fruit (*P. edulis*) as an example, the AMs of juvenile plants give rise to a vegetative meristem, those in adult vegetative plants produce a tendril next to a vegetative meristem, and finally, adult reproductive plants form, in addition to the vegetative meristem, an AM that divides into two primordia to form tendrils and flowers simultaneously (Ulmer and Macdougal, 2004; Dornelas et al., 2006; Cutri et al., 2013). Passion fruit species evolved in ecosystems in which competition for light is the norm, due to dense vegetation (Ulmer and Macdougal, 2004). Therefore it can be considered an adaptive advantage the ability to climb on other plants in order to reach the top of the canopy. *Passiflora* species endure a very short juvenile stage (about eight plastochrons) under the canopy shadows and after transitioning to the adult stage, tendrils are produced by lateral axillary meristems (Cutri et al., 2013). The production of flowers is repressed in *P. edulis* plants under shaded conditions, and thus tendrils allow the plant to climb to the top of the canopy where flowers can develop. According to these observations, flowers are formed only after tendrils are formed and they share a common ontogenetic origin (Cutri et al., 2013; Sousa-Baena et al., 2018). The number and position of flowers formed from the axillary meristems diverge among *Passiflora* species. Cutri et al. (2013) showed by comparing different *Passiflora* species under distinct environmental conditions that a great ontogenetic plasticity exists that is normally restrained by genetic, hormonal and environmental constraints. Therefore we postulate that what appears to be a species-specific program regulating the fate of the *Passiflora* lateral axillary meristems, is in great part due to a balance of the expression patterns of FT/TFL1 orthologs in passion fruits.

## Conclusion

The balance between *FT*/*TFL1* ortholog genes is important for adaptation of plants to diverse environmental conditions. It is notable that domestication of several wild and exotic species into agronomic cultures with specific growth habits results from a selection of the differential balance between *FT*/*TFL1*. Thus, studies characterizing the interaction between these genes become an important tool for breeding programs of plants of commercial interest, since the ability to modulate plant size might allow increasing planting density, facilitate fruit harvest, and increase crop productivity, among other agronomic benefits. Considering that passion fruit AMs are predicted to be more complex in comparison with AMs in other species, we propose passion fruit as an appropriate model to study the *FT*/*TFL1* balance in order to understand how AM modulation gives rise to different structures.

## Data Availability

The datasets generated for this study are available on request to the corresponding author.

## Author Contributions

TM and MD designed the initial manuscript. TM wrote the initial draft of the manuscript and conceived the figure. TM, MD, and AM contributed reviewing and discussing the manuscript to produce its final version.

### Conflict of Interest Statement

The authors declare that the research was conducted in the absence of any commercial or financial relationships that could be construed as a potential conflict of interest.
